# Role of Antihypertensives in End-Stage Renal Disease: A Systematic Review

**DOI:** 10.7759/cureus.27058

**Published:** 2022-07-20

**Authors:** Rizwan Rabbani, Edva Noel, Suzanne Boyle, Hema Balina, Sabahat Ali, Bolajoko Fayoda, Waqas Ahmad Khan

**Affiliations:** 1 Nephrology, Temple University Hospital, Philadelphia, USA; 2 Family Medicine, University College of Medicine and Dentistry, Lahore, PAK

**Keywords:** dialysis, renal insufficiency, hemodialysis, antihypertensive drugs, chronic renal failure

## Abstract

The primary goal of this research is to identify the factors of intradialytic hypertension in hemodialysis patients and stabilize blood pressure (BP) even without antihypertensive medicines. There are various treatment alternatives for lowering BP in these patients, many of which do not require extra pharmacological therapy (e.g. long, slow hemodialysis; short, daily hemodialysis; nocturnal hemodialysis; or, most effectively, dietary salt and fluid restriction in addition to the reduction of dialysate sodium concentration). These parameters provide good monitoring of BP, even with previously diagnosed hypertension. The adjustment of the extracellular volume with a low incidence of intradialytic hypotensive episodes is the most plausible explanation for this outcome.

We did a systematic evaluation of all published articles since 1994 to evaluate antihypertensive drug outcomes in hemodialysis patients. All articles were searched in the English language using PubMed and Google Scholar databases. The screening techniques, study selection, data extraction procedures, and risk evaluation of bias were done using specified criteria and overseen by one of the senior writers with the application of quality assessment tools to the final articles. Data were searched using regular and MeSH (Medical Subject Headings) keywords. Although substantial developments have emerged in the medical field, there is still a significant knowledge gap in the sector, particularly when it comes to BP guidelines and therapy choices for hypertensive hemodialysis patients. Until additional data are available, we should treat hypertension in hemodialysis with the use of active pursuit of euvolemia using dry weight probing and reduction of salt excess.

## Introduction and background

In addition to removing excess fluid and filtering waste products, hemodialysis (HD) replaces some of the basic functions of healthy kidneys. Because of this filtering effect, patients on HD should use caution when taking certain medications, such as antihypertensive drugs [1]. People with chronic kidney disease (CKD) may experience hypertension caused by various mechanisms, including an increased vascular volume, increased peripheral vascular resistance, increased activity of the renin-angiotensin-aldosterone system, and decreased renal vasodilator prostaglandins [2]. Managing blood pressure (BP) effectively in patients with end-stage renal disease (ESRD) can be challenging. However, symptomatic hypotension may require early discontinuation of HD before achieving optimal treatment goals because HD removes excess fluid and reduces circulating volume. Are antihypertensive drugs withheld to prevent hypotension that may result from fluid loss during HD? This issue does not have a one-size-fits-all solution.

It has recently been reported that intra- and post-HD hypertension are independent risk factors for mortality in hypertensive HD patients. Over the last few years, numerous studies have examined the mechanisms of intradialytic hypertension and its management. Cardiovascular (CV) risks are higher in HD patients with hypertension. The level to which BP should be reduced or when and where to measure BP in such patients is not guided by any data. More than 72 million Americans have been diagnosed with hypertension, and 13% of them have stage 3 or higher CKD [3,4]. As the second most common cause of ESRD, hypertension is responsible for more than two-thirds of dialysis patients today [5,6].

Pre-HD systolic blood pressure (SBP) overestimates ambulatory blood pressure monitoring (ABPM) values, according to a meta-analysis of BP assessed at different times over HD [7]. SBP of pre-HD patients increases in response to fluid volume increases, antihypertensive medications are withheld before treatment, and measurements are not standardized. Despite being closer to 24-hour ABPM readings, post-HD measurements underestimate ambulatory values [7]. ABPM values provide more representative data than any single point in ESRD because they take multiple BP readings during the day, with special attention paid to early morning BP when CV risks are at their greatest. In this article, we would like to find out the causes of hypertension, the acceptable range of BP, and the circumstances in which antihypertensives can be withheld in hypertensive HD patients.

## Review

Methods

A systematic review of all published studies since 1994 was conducted to determine the effects of antihypertensive drugs in HD patients. All studies were consulted in English through PubMed and Google Scholar. The data were retrieved with the help of regular and MeSH (Medical Subject Headings) keywords. Two different authors have analyzed all the retrieved studies separately by considering all the predetermined criteria. Every study has been checked according to the Assessment of Multiple Systematic Reviews (AMSTAR) quality tool.

Regular and MeSH Keywords

Chronic renal failure: Chronic renal failure OR kidney insufficiency OR compromised renal function OR end-stage renal disease OR ("kidney failure, chronic/complications"[MeSH] OR "kidney failure, chronic/drug therapy"[MeSH] OR "kidney failure, chronic/pharmacology"[MeSH] OR "kidney failure, chronic/physiology"[MeSH] OR "kidney failure, chronic/physiopathology"[MeSH] OR "kidney failure, chronic/therapy"[MeSH]).

Dialysis: Dialysis OR hemodialysis OR peritoneal dialysis OR removal of waste OR kidney failure OR "renal dialysis/adverse effects"[MeSH] OR "renal dialysis/pharmacology"[MeSH] OR "renal dialysis/therapeutic use"[MeSH] OR "renal dialysis/therapy"[MeSH]).

Antihypertensive drugs: Alpha blocker OR beta blocker OR ACE inhibitor OR calcium channel blocker OR ("antihypertensive agents/adverse effects"[Mesh] OR "antihypertensive agents/chemical synthesis"[MeSH] OR "antihypertensive agents/pharmacokinetics"[MeSH] OR "antihypertensive agents/pharmacology"[MeSH] OR "antihypertensive agents/physiology"[MeSH] OR "antihypertensive agents/therapeutic use"[MeSH] OR "antihypertensive agents/therapy"[MeSH] OR "antihypertensive agents/toxicity"[MeSH]).

Inclusion and Exclusion Criteria

Five inclusion criteria have been implemented to collect the data: (i) past 28 years of studies conducted with emphasis on newer studies; (ii) only patients who were on HD; (iii) studies exclusively on humans; (iv) hypertensive patients; and (v) studies written in the English language. Exclusion criteria included young patients < 25 years and patients on peritoneal dialysis.

Quality Evaluation

**Figure 1:**
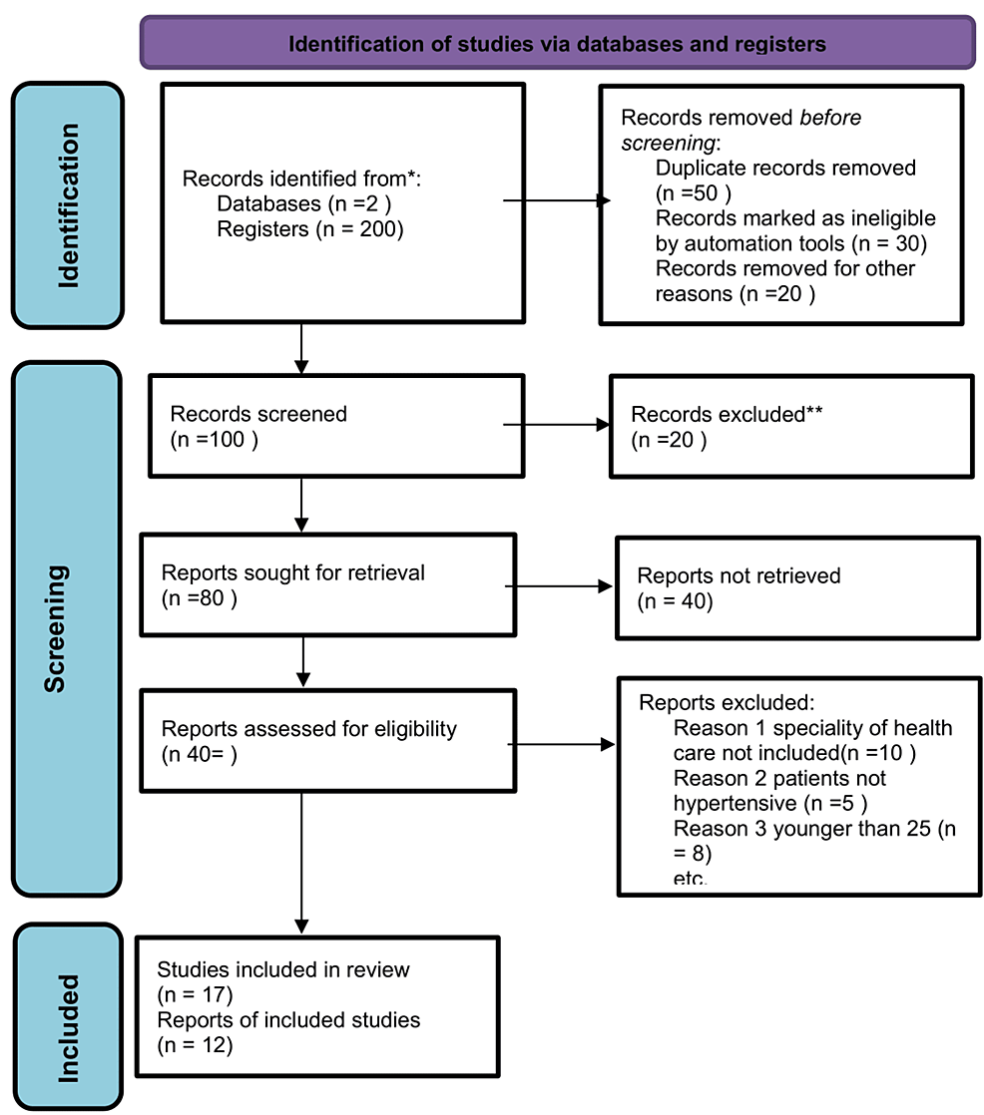
PRISMA flow diagram PRISMA: Preferred Reporting Items for Systematic Reviews and Meta-Analyses.

We used Newcastle-Ottawa Scale for cohort and case-control studies. The National Institutes of Health (NIH) quality evaluation tool was used to conduct a cross-sectional study. Additionally, the quality of systemic reviews/meta-analyses was evaluated by AMSTAR-2. All low-quality items were overlooked, and only medium and high-quality items were incorporated. The Preferred Reporting Items for Systematic Reviews and Meta-Analyses (PRISMA) flow diagram giving a brief overview of our data search can be seen in Figure 1.

Discussion

There are no BP targets defined for patients undergoing HD, but most clinicians agree that intervention is warranted in cases of high BP. Until more definitive data are available, clinicians suggest keeping HD BP in the range of 130-160 mmHg/80-100. It is not recommended that further BP decreases are undertaken at this time. Katzarski et al. [8] showed that by controlling extracellular fluid, BP can be controlled in patients undergoing long HD sessions. Strategies should prioritize the management of salt and water balance first, since regulating extracellular fluid volume leads to better control of BP. Decreased sodium intake [9], increased ultrafiltration, and closer attention to the sodium dialysate prescription (describe additional below) are precautionary measures to maintain BP without antihypertensive drugs. In observational studies and clinical trials, longer and/or more frequent dialysis results in better BP control [10,11].

**Figure 2:**
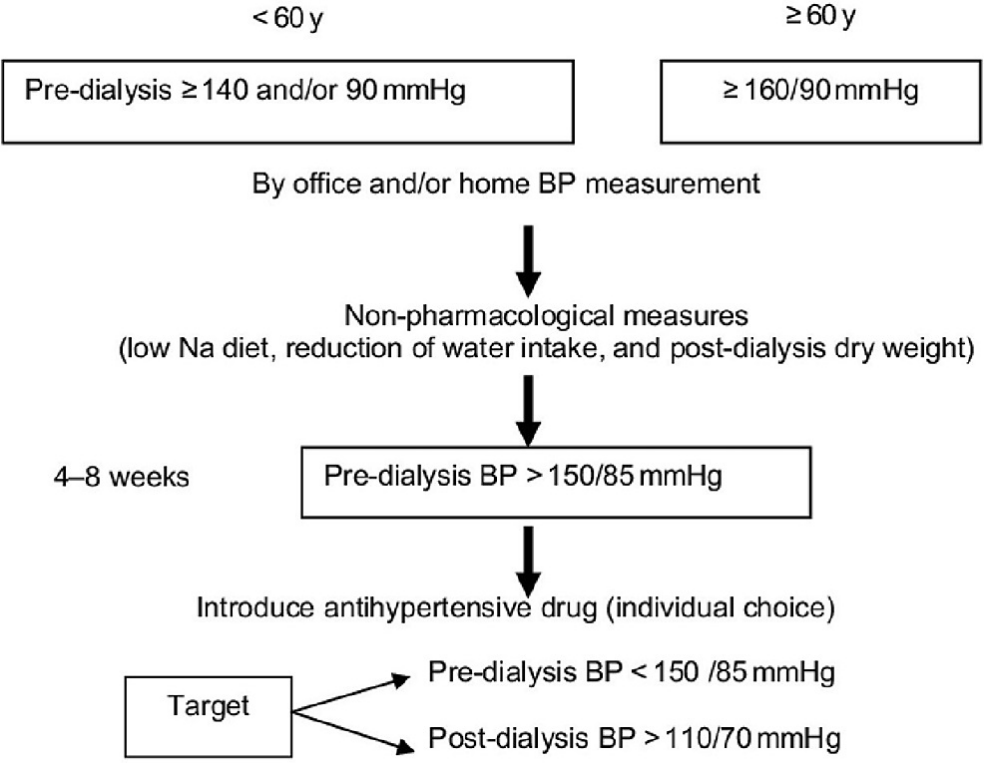
Diagnostic range of hypertension in hemodialysis patients BP: blood pressure.

It is very hard to establish a specific range for hypertension in ESRD patients. After studying all the literature, we have concluded a specific range for hypertension. In young patients, if BP is equivalent to 140/90, then they will not be considered hypertensive patients. In patients aged more than 60 years, if the BP range is more than 160/90, then they will be considered hypertensive. Everyone agrees with non-pharmacological measures before starting antihypertensive drugs [12]. The range of BP is described in Figure 2.

**Figure 3:**
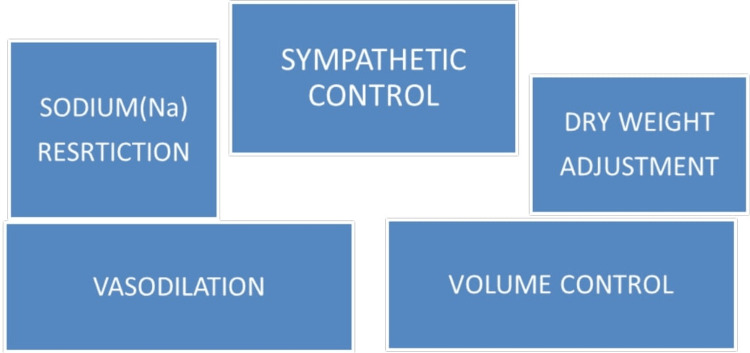
Blood pressure regulation factors in hemodialysis patients

Several mechanisms contribute to BP control including volume optimization [12], vasodilation (endothelium-dependent and independent), and the reduction of sympathetic tone (Figure 3) [13]. The majority of patients undergoing long-term HD do not require medication for hypertension.

Influence of hydration status

Dionisio et al. have found a correlation between interdialytic ambulatory BP and hydration state. Other studies have also discovered a correlation between hydration status and intradialytic BP measurements [14]. The interdialytic BP of HD patients appears to be controlled by the hydration status of the body. Considering ambulatory BP and hydration state, we conclude that these variables are significantly correlated. Since SBP is more significantly correlated with overhydration than diastolic blood pressure (DBP), SBP is more likely to be affected by overhydration. Comparing the BP values of those with and without antihypertensive medication, it is interesting to note that the former group had higher BP. This observation is of great significance since it suggests that BP can be controlled without antihypertensive medication, probably by more precisely prescribing ultrafiltration [15].

The short HD schedules with high ultrafiltration are associated with frequent hypotensive episodes and weight gain instead of achieving the desired dry weight: the patient becomes overweight and requires higher ultrafiltration and antihypertensive medication. As an alternative to antihypertensive medication, long dialysis and slow ultrafiltration can decrease hypotensive episodes and allow the patient to maintain a normal dry weight and control their BP without taking antihypertensive medication. It appears that optimizing hydration status is the best way to improve HD patients' BP values and survival rate.

Effect of volume control

High BP is frequent and difficult to control in the HD population, explaining the high mortality rate in this population [16,17]. Other risk factors for cardiovascular disease are also prevalent in this patient group. Patients requiring a total ultrafiltration volume greater than 2.5 kg may have an elevation in presystolic and prediastolic BPs. Higher pre- and post-SBP are associated with diabetes mellitus, older age, and intake of more antihypertensive medications. [18] Continuing reflection favors the maintenance of normal BP values in the general population as well as in HD patients. A low salt diet and efficient control of volume are key factors for maintaining BP in HD patients [19]. Drugs like angiotensin-converting enzyme (ACE) inhibitors and beta-blockers are not only used for controlling left ventricular hypertrophy but also for sympathetic overactivity.

Restriction of sodium

Because of salt and volume management, 95% of patients in Laurent's [20] investigations in Tassin (France) had normal BP without medication in the first weeks of extended dialysis therapy. Hypertension can be controlled without prolonging dialysis duration by restricting salt in the diet and lowering sodium in the dialysate. In conjunction with a NaCl-restricted diet of no more than 6 g/day, the sodium content in the dialysate was gradually reduced from 140 to 135 mmol/L at a rate of 1 mmol/L every three to four weeks in the above study. It is permissible to withhold antihypertensive treatment during the interdialytic interval if BP remains normal and does not exceed 160/95 mmHg immediately before the next dialysis session [21]. In a six-week pilot study, the authors found reduced dialysate sodium concentration from 140 to 136 mmol/L but found no significant changes in pre-dialysis SBP or DBP [22]. These patients, on the other hand, were kept on a sodium-restricted diet. This may clarify the failure of further decrease in BP by reducing dialysate sodium concentration [23].

Sodium overload raises intracellular sodium and calcium concentrations, causing an increase in vascular smooth muscle tonicity. Sodium reduction, on the other hand, may reverse this action, resulting in a fall in BP [24].

The idea of dialysis adequacy has evolved and has been shaped by clinical experience, according to Weiner et al. We have long moved away from focusing solely on Kt/V (a parameter used to determine the efficacy of an HD session, it shows the clearance of potassium K in a given patient with a specific volume of distribution), and now dialysis adequacy is a multifaceted approach that includes several domains aimed at restoring internal milieu homeostasis and improving the patient experience. Restoration of extracellular volume, adequate BP management, and hemodynamic stability in HD patients remain elusive even after more than 50 years of the invention of renal replacement therapy [25].

Dialysis patients' sodium mass balance has progressed from a clinical dry weight approach to fluid management with diagnostic equipment to a new age of direct dialysis sodium and water control utilizing modern technology and analytics. Three primary components of total body sodium have been identified, which include circulating and systemically active sodium with its osmotic action, a sodium reservoir in the bones that exchanges slowly, and a reservoir of sodium in the interstitium of skin and muscles. Through local hypertonicity and sodium clearance processes, this pool is engaged in cell and biologic functions.

For a focused and individualized approach, new techniques for monitoring and regulating salt and water in dialysis patients are essential. By enhancing hemodynamic stability and dialysis tolerance, emerging advanced knowledge has the potential to enhance patient outcomes. Moreover, this is an example of translational medicine in which novel discoveries (e.g. tissue sodium buildup) are translated into creative tools (e.g. ^23^NaMRI and dialytic sodium balance) to enhance patient benefits and awareness [26]. Extensive prospective outcome studies are required for this suggested integrated strategy for salt and fluid control.

Dry weight

Dry weight is defined as the post-dialysis weight at which the patient maintains a normotensive state without the need for antihypertensive medication until the next dialysis or as the body weight after dialysis beyond which further reduction triggers hypotension. Biochemical markers, bioimpedance analysis, bioimpedance spectroscopy, and vena cava diameter are used to determine dry weight. Atrial natriuretic peptide (ANP) and cyclic guanidine monophosphate are two biochemical indicators.

ESRD is a catabolic condition that leads to nephron loss, which causes weight gain with an unexpected reduction of lean body mass over time. Extracellular volume expansion and salt retention are caused by gradual nephron loss. Weight increase occurs as a result, while lean body mass is unexpectedly reduced.

Hypervolemia occurs in chronic renal disease because of a decrease in glomerular filtration rate, which results in positive sodium balance and extracellular fluid expansion. This is coupled with excessive dietary sodium and fluid intake on many occasions. There is also secretion of ouabain-like inhibitors of Na,K-ATPase, which lead to elevation of intracellular calcium, causing an increase in vascular resistance.

Sympathetic control

Overactivity of the renin-angiotensin system is another reason for hypertension in dialysis patients. It arises as a result of CKD-related localized renal ischemia and scarring, which leads to increased renin release and increased systemic vascular resistance. Increased sympathetic activity, which is caused by higher levels of angiotensin 2, also contributes to higher vascular resistance and systemic BP. It is also linked to asymmetric dimethylarginine, whose function is not fully known. Uremia, higher endothelin-1, erythropoietin administration for the treatment of CKD anemia, hyperparathyroidism, and elevated pulse pressure are among the other causes of hypertension in dialysis patients. Uremia, which is caused by CKD, triggers a neural reflex that stimulates the brainstem's cardiovascular centers. As a result of reduced renal clearance, endothelin 1, a strong vasoconstrictor, is accumulated inside the body.

In individuals with chronic renal failure, erythropoietin given subcutaneously to treat chronic anemia raises BP by 10 mmHg. In individuals with CKD, hyperparathyroidism is another cause of high BP. Calcification of arterial trees accelerates vascular resistance, which leads to an increase in pulse pressure, which plays a role in the pathogenesis of hypertension. Because of their ineffectiveness, diuretics are rarely used as antihypertensive medication. Antihypertensive medication is chosen based on concomitant comorbidities, patient demographics, risk profile, and lifestyle.

The study characteristics of 12 studies are presented in Table 1. In every study, some interventions have been used to control BP in hypertensive patients.

**Table 1:** Blood pressure control by using different interventions without antihypertensive drugs (study characteristics) EBC: electrolyte balancing control.

Authors	Study type	No. of patients	Intervention	Outcome
Agarwal et al. [7]	Experimental	50	Dry weight reduction	Change in Interdialytic arterial blood pressure decreases hypovolemia
Katzarski et al. [8]	Clinical trial	112	Control of extracellular fluid	Blood pressure will be controlled in long hour hemodialysis sessions
Kayikcioglu et al. [9]	Cross-sectional	190 + 204	Salt restriction and stop antihypertensive drugs	Decrease hypotension in hemodialysis patients
Luik et al. [12]	Clinical trial	21	Dialysis time increase	During dialysis, systolic blood pressure decreases
Dionisio et al. [15]	Clinical trial	45	Ambulatory blood pressure monitoring	Showed a significant relationship with hydration status
Mailloux et al. [17]	Systematic review		Control extracellular fluid, dry weight, and salt restriction	Target pre-dialysis systolic blood pressure greater than 140/90
Ozkahya et al. [22]	Experimental	67 hemodialysis patients	Reduction of salt intake > 100 mmol/g	Blood pressure decreased from 173 ± 17/102 ± 9 to 139 ± 18/86 ± 11 mmHg
Laurent [20]	Experimental	110	Switch to long dialysis from 3 months without taking antihypertensive drugs	Mean arterial pressure reduced from 116 mmHg to 99 mmHg
Krautzig et al. [21]	Clinical trial	8 hypertensive patients	Dialysate Na from 140 to 130 mmol/l	Presystolic and diastolic blood pressure decreased significantly
Kooman et al. [23]	Random trial	5 of 120 patients	Dialysate 140 mEq/L for 6 weeks	No significant change in blood pressure Kt/v prognostic value for hemodialysis
Weiner et al. [25]	Observational and retrospective	Elderly and above 50	Fluid intake and volume control	Blood pressure control without antihypertensive medication
Canaud et al. [26]	Pilot study	50 and above	EBC (microsensor) used in dialysate inlet and outlet streams	Precise sodium and water balance helps in the control of blood pressure

Limitations and further research

There is a drawback in this study in terms of patient selection with comorbidities. Furthermore, we have not taken into account ethnic, sex, or nutrition disparities in the population. More research is needed to determine the true etiology of hypertension and then treat it appropriately.

## Conclusions

Intradialytic hypertension is a prevalent condition in the dialysis population (prevalence: 5%-15%). This variant indicates a significant chance of mortality. However, it is unclear whether this increased risk is due to per-session intradialytic hypertension or baseline systemic hypertension as determined by ABPM, a question that needs to be addressed further in future investigations. This change is caused by volume and salt overload, endothelial dysfunction, and the reticular activating system (RAS) and sympathetic nervous system (SNS) overactivity. This is the main reason hypertensive patients who were taking antihypertensive drugs have experienced hypotension episodes during HD. The management of intradialytic hypertension and hypotension necessitates a thorough implementation of current guidelines for the management of hypertension in HD patients. Most nephrologists believe to withhold antihypertensive drugs before dialysis to avoid hypotension episodes as a certain range of hypertension is less dangerous than intradialytic hypotension. Controlling sodium and volume overload, using prolonged or frequent dialysis to alleviate excessive intradialytic RAS and SNS activation, the wise use of specific antihypertensive classes, most commonly used calcium channel blockers, alpha-blockers, ACE inhibitors, and angiotensin receptor blockers to aliskiren and minoxidil, or even renal denervation in patients truly unresponsive to multiple drug therapy and dialysis optimization are all options until more specific evidence targeting mechanistic pathways of intradialytic hypertension becomes available.
